# Impact of the ENSP eLearning platform on improving knowledge, attitudes and self-efficacy for treating tobacco dependence: An assessment across 15 European countries

**DOI:** 10.18332/tid/120188

**Published:** 2020-05-07

**Authors:** Charis Girvalaki, Sophia Papadakis, Enkeleint A. Mechili, Katerina Nikitara, Andrey Demin, Antigona C. Trofor, Arben Lila, Arusyak Harutyunyan, Aurela Saliaj, Deska Dimitrievska, Francisco Rodriguez Lozano, George Bakhturidze, Javier Ayesta, Krzysztof Przewoźniak, Maria Sofia Cattaruzza, Marija Zdraveska, Mihaela Lovše, Biljana Kilibarda, Otto Stoyka, Panagiotis Behrakis, Pierre Bizel, Polina Starchenko, Shkumbin Spahija, Cornel Radu-Loghin, Constantine I. Vardavas

**Affiliations:** 1 European Network for Smoking and Tobacco Prevention, Brussels, Belgium; 2 Medical School, University of Crete, Heraklion, Greece; 3 Division of Prevention and Rehabilitation, University of Ottawa Heart Institute, Ottawa, Canada; 4 Faculty of Medicine, University of Ottawa, Ottawa, Canada; 5 Department of Health Care, Faculty of Public Health, University ‘Ismail Qemali’ Vlore, Vlora, Albania; 6 Institute of Leadership and Healthcare Management, I.M. Sechenov First Moscow State Medical University, Moscow, Russia; 7 University of Medicine and Pharmacy ‘Grigore T. Popa’ Iasi, Iasi, Romania; 8 AER PUR Romania, Bucharest, Romania; 9 Kosovo Advocacy and Development Center, Pristina, Kosovo; 10 Turpanjian School of Public Health, American University of Armenia, Yerevan, Armenia; 11 Macedonian Respiratory Society, Skopje, North Macedonia; 12 Comité Nacional de Prevención del Tabaquismo, Madrid, Spain; 13 Tobacco Control Alliance of Georgia, Tbilisi, Georgia; 14 University of Cantabria, Santander, Spain; 15 Foundation ‘Smart Health – Health in 3D’, Warsaw, Poland; 16 Maria Sklodowska-Curie National Research Institute of Oncology, Warsaw, Poland; 17 Department of Public Health and Infectious Diseases, Sapienza University, Rome, Italy; 18 Società Italiana di Tabaccologia (SITAB), Rome, Italy; 19 Slovenian Coalition for Public Health, Environment and Tobacco Control, Maribor, Slovenia; 20 Institute of Public Health of Serbia ‘Dr Milan Jovanović Batut’, Belgrade, Serbia; 21 Kyiv Health Center, Kyiv, Ukraine; 22 George D. Behrakis Research Laboratory, Athens, Greece; 23 Hellenic Cancer Society, Athens, Greece; 24 Wallionie Tabac Prevention, Brussels, Belgium

**Keywords:** evidence-based strategies, healthcare professionals, eLearning, smoking cessation

## Abstract

**INTRODUCTION:**

In 2018, the European Network for Smoking Cessation and Prevention (ENSP) released an update to its Tobacco Treatment Guidelines for healthcare professionals, which was the scientific base for the development of an accredited eLearning curriculum to train healthcare professionals, available in 14 languages. The aim of this study was to evaluate the effectiveness of ENSP eLearning curriculum in increasing healthcare professionals’ knowledge, attitudes, self-efficacy (perceived behavioral control) and intentions in delivering tobacco treatment interventions in their daily clinical routines.

**METHODS:**

We conducted a quasi-experimental pre-post design study with 444 healthcare professionals, invited by 20 collaborating institutions from 15 countries (Albania, Armenia, Belgium, Italy, France, Georgia, Greece, Kosovo, Romania, North Macedonia, Russia, Serbia, Slovenia, Spain, Ukraine), which completed the eLearning course between December 2018 and July 2019.

**RESULTS:**

Healthcare professionals’ self-reported knowledge improved after the completion of each module of the eLearning program. Increases in healthcare professionals’ self-efficacy in delivering tobacco treatment interventions (p<0.001) were also documented. Significant improvements were documented in intentions to address tobacco use as a priority, document tobacco use, offer support, provide brief counselling, give written material, discuss available medication, prescribe medication, schedule dedicated appointment to develop a quit plan, and be persistent in addressing tobacco use with the patients (all p<0.001).

**CONCLUSIONS:**

An evidence-based digital intervention can be effective in improving knowledge, attitudes, self-efficacy and intentions on future delivery of tobacco-treatment interventions.

## INTRODUCTION

Tobacco use is the most significant threat to European Public Health and directly associated with morbidity and mortality^1^. More than a quarter (26%) of Europeans still smoke^1^. Smoking cessation is one of the main strategies suggested by the World Health Organization’s (WHO) MPOWER package against the tobacco epidemic, presenting the first comprehensive worldwide analysis of tobacco use and control efforts^2^. The WHO Framework Convention on Tobacco Control (WHO FCTC) Article 14 and its implementation guidelines call on its Parties to ‘facilitate accessibility and affordability for treatment of tobacco dependence’^2^. Healthcare professionals have a key role to play in supporting tobacco treatment delivery, one of the targets of the WHO MPOWER package and FCTC^3^.

Training is an integral part of supporting tobacco treatment delivery and has been shown to increase the likelihood of healthcare professionals delivering evidence-based tobacco treatment. Despite this, only a fraction of European healthcare professionals have completed training as part of their formal undergraduate education or as part of continuing medical education^4^. Recent reviews show that there is a gap in the use of advanced computer-based medical education approaches on tobacco treatment training for healthcare professionals, and there is a need to support the training/education of healthcare professionals in behavioral change in Europe^4^.

In 2018, the European Network for Smoking Cessation and Prevention (ENSP) released an update to its tobacco treatment guidelines for healthcare professionals^5^. The guidelines summarize the latest evidence and provide recommendations for healthcare professionals (www.elearning-ensp.eu). An eLearning curriculum was developed to train healthcare professionals in the guideline recommendations, available in 14 languages.

This study reports on the effectiveness of an accredited eLearning curriculum that aimed to increase healthcare professionals’ knowledge, and to change attitudes, self-efficacy (perceived behavioral control) and intentions in delivering tobacco-treatment interventions in their daily clinical life.

## METHODS

In the context of EPACTT-Plus, collaborating institutions from 15 countries (Albania, Armenia, Belgium, Italy, France, Georgia, Greece, Kosovo, Romania, North Macedonia, Russia, Serbia, Slovenia, Spain, Ukraine) worked to develop an accredited eLearning course on Tobacco-Treatment Delivery available at http://elearning-ensp.eu/. This five-module eLearning training course was accredited by the European Accreditation Council for Continuing Medical Education (EACCME) for 2 CME credits. Ajzen’s Theory of Planned Behavior (TPB) was used to guide intervention design. The TPB incorporates both social influences and personal factors as predictors, specifying a limited number of psychological variables that can influence behavior such as attitude, subjective norms, perceived behavioral control (PBC), and intention^6^.

### eLearning curriculum

The curriculum includes five different training modules as well as case studies, quizzes, and interactive content. The course was developed in Moodle, and is available at www.elearning-ensp.eu. At the end of each module, a knowledge assessment is completed by users.

#### Module 1

This module is entitled ‘Nicotine Addiction - Why people smoke?’ and discusses why it is so difficult for individuals to quit smoking. The physical and psychological aspects of tobacco use are reviewed, as well as the pathophysiology of nicotine addiction and the associated withdrawal symptoms and cravings that can make quitting challenging. In addition are discussed the connections between daily routines, triggers to smoke and mood in maintaining tobacco use.

#### Module 2

This module is entitled ‘How to help your patients quit smoking’. The important role of health professionals in supporting cessation among patients who smoke as a clinical priority is reviewed as well as a brief overview of the 5As model for integrating tobacco dependence treatment into clinical settings. The 5As include: ‘Ask’ patients about smoking status; ‘Advice’ them to quit smoking; ‘Assess’ their readiness to quit smoking; ‘Assist’ them with making a quit attempt; and ‘Arrange’ a follow-up meeting.

#### Modules 3 and 4

The behavioral counselling content is divided into two parts, Modules 3 and 4. In Module 3, the role of behavioral counselling as a treatment for supporting tobacco users with quitting is discussed as well as evidence-based counselling strategies designed for individuals ready to quit smoking in order to increase their likelihood of successfully quitting. In Module 4, key behavioral strategies used to enhance motivation among patients who are not ready to quit smoking include motivational interviewing and smoking reduction approaches. These two modules also include examples of how to deliver behavioral-change techniques as an active learning method.

#### Module 5

This module provides training on pharmacological treatment as a fundamental component of treating tobacco use. In this module, first-line quit smoking medications are discussed including Nicotine Replacement Therapy, Bupropion, Varenicline and their appropriate use in clinical practice in order to maximize success with smoking cessation among patients. A brief overview of other quit-smoking medications that are emerging as promising therapies are also presented to learners. Several interactive case studies are embedded in this module as an active learning method.

### Adaptation and translation of the material

In order to adapt and translate the eLearning content for each participating country, local champions were engaged. The training program was adapted and translated into 14 different languages (Albanian, Armenian, English, French, Georgian, Greek, Italian, Macedonian, Romanian, Russian, Serbian, Slovenian, Spanish, Ukrainian) and released in 2018.

### Evaluation of the training curriculum

During the recruitment period, an email to relevant healthcare professionals was sent from collaborating institutes in each country, to invite them to take the courses. A cut-off of 30 participants per language was set as a limit for the evaluation, while the complete duration of the sessions was about two and a half hours of online training.

A pre-post assessment was embedded in the structure of the eLearning courses. During the pre-assessment, and following the provision of consent, participants reported their demographic characteristics, current practices in delivering tobacco treatment practices (5As delivery with 9 components), their normative beliefs (5 components), attitudes (6 components), self-efficacy in delivering tobacco treatment interventions (5 components), perceived importance in providing these interventions (1 component), and intentions (9 components). During the post-assessment, the same variables were assessed to document changes. In addition to the above, before and after each module, participants completed a knowledge assessment. A score of 80% or higher was required to pass the test. The tools used to evaluate the training curriculum have been previously tested in several studies conducted from members of our team^7-11^.

### Statistical analysis

Quantitative variables that followed normal distributions are presented as means and standard deviations (SD). Quantitative variables that did not follow a normal distribution and ordinal variables are presented as median and interquartile range (non-parametric methods). Qualitative variables are expressed as frequencies and percentages. In the case of related samples (paired comparisons), paired t-test (when the variable was continuous and followed a normal distribution) and Wilcoxon signed-rank test (when the variable did not follow a normal distribution or was an ordinal variable) were used. In this case, results are presented as the median difference, 95 % confidence interval for the difference and IQR 25–75, while a z-test was used for the difference of the median between the pre-test ranks and the post-test ranks. Statistical significance was set at 0.05. All analyses were performed in IBM SPSS statistical software package version 23.0.

## RESULTS

From December 2018 to July 2019, a total of 444 healthcare professionals were enrolled and completed the course. Table 1 presents the characteristics of participating healthcare professionals and their practices. The majority of the participants were female (73.4%), under 30 years of age (43.6%), working in the public sector (73.9%) and urban settings (81.9%). Most of the participants (68.9%) had not participated in smoking cessation training in the past, while 32.2% reported being smokers (67.8%).

**Table 1:** Characteristics of healthcare professionals who participated in the ENSP eLearning for treating tobacco dependence

*Variables*	*n*	*%*
**Gender**		
Male	118	26.6
Female	326	73.4
**Age** (years)		
<30	193	43.5
30–39	85	19.1
40–49	93	20.9
50–59	48	10.8
60–69	24	5.4
≥70	1	0.2
**Practice setting**		
Private	116	26.1
Public	328	73.9
**Geographical location**		
Urban	363	81.9
Suburban	45	10.2
Rural	35	7.9
**Employment status**		
Part-time	80	18.0
Full-time	364	82.0
**Previous training in smoking cessation**		
Yes	138	31.1
No	306	68.9
**Health care provider smoking status**		
Smoker	33	7.4
Ex-smoker	110	24.8
Non-smoker	301	67.8
**Practice supporting facilities for treating tobacco dependence**		
Process to screen and document smoking status of patients	242	56.5
Self-help materials for smokers	120	28.0
Consult forms to guide through quit smoking counselling interventions	66	15.4

Table 2 depicts the recorded changes in healthcare professionals’ self-reported knowledge before and after the completion of each module of the eLearning program. Statistically significant changes were documented for all knowledge questions, except one, of Module 1, Module 2, and behavioral counselling Modules 3 and 4 (p<0.001). A significant increase in participant’s knowledge regarding pharmacotherapy (Module 5) was also recorded. For example, there was an increase in knowledge regarding the safety of the NRT (45.7% vs 97.0%; p<0.001) and the most common side effect of Varenicline (68.5% vs 96.2%; p<0.001).

**Table 2:** Proportion of trainees who responded correctly to knowledge assessment at the pre- and post-assessments

*Variables*	*Pre n (%)*	*Post n (%)*	95% Cl*	p**
**Knowledge^a^**				
**Nicotine addiction Module 1**				
For most smokers, tobacco use is…	186 (42.3)	394 (89.5)	0.420–0.525	<0.001
Nicotine is as addictive as other drugs such as heroin or cocaine.	350 (79.5)	424 (96.4)	0.129–0.208	<0.001
Which of the following are common withdrawal symptoms experienced when someone quits smoking?	369 (83.9)	432 (98.2)	0.107–0.179	<0.001
How soon may someone begin to experience withdrawal symptoms when they go without a cigarette?	149 (33.9)	386 (87.7)	0.482–0.595	<0.001
Which of the following are not true about nicotine withdrawal symptoms?	192 (43.6)	373 (84.8)	0.356–0.466	<0.001
What proportion of smokers report night wakening or difficulty sleeping after they quit?	230 (52.3)	417 (94.8)	0.373–0.477	<0.001
The majority of patients who quit smoking experience irritability, restlessness, and depression?	332 (75.5)	420 (95.5)	0.157–0.243	<0.001
Which of the following are not true about people who are fast nicotine metabolizers?	311 (70.8)	419 (95.4)	0.198–0.294	<0.001
What are the most common reasons people return to smoking in the first few weeks after quitting?	336 (76.4)	428 (97.3)	0.166–0.252	<0.001
People who quit smoking will have more, less, or the same amount of stress?	185 (42.0)	399 (90.5)	0.434–0.537	<0.001
**How to help patients quit Module 2**				
Tobacco treatment should be delivered with the same rigor and clinical importance as any other major chronic risk factor.	418 (94.6)	436 (98.6)	0.019–0.062	<0.001
A clinician’s advice to quit is not very effective in motivating patients to quit.	327 (74.0)	425 (96.2)	0.176–0.268	<0.001
Advice to quit smoking should be…	348 (78.7)	429 (97.1)	0.143–0.223	<0.001
Offering patients your support with quitting has been shown to increase patient motivation to quit.	409 (92.5)	439 (99.3)	0.042–0.094	<0.001
If a patient informs you they are not ready to quit, what should you do?	396 (89.6)	437 (98.9)	0.064–0.122	<0.001
Which of the following questions are important pieces of information gathered as part of the smoking history? (check all that are correct)	382 (86.4)	435 (98.4)	0.086–0.153	<0.001
Which of the following are important pieces of information to discuss with patients when developing a motivational intervention or personalized quit plan?	383 (86.8)	434 (98.4)	0.082–0.149	<0.001
Patients that have higher levels of nicotine dependence can be assessed by…	262 (59.3)	390 (88.2)	0.236–0.344	<0.001
For patients who report they are interested in quitting smoking, evidence has shown that the most effective evidence-based treatments are…	324 (73.3)	418 (94.6)	0.170–0.255	<0.001
It is recommended that follow-up be scheduled 2–8 weeks, following the patient’s initial consultation, to review progress and response to therapy and make any adjustments to the plan.	371 (84.3)	432 (98.2)	0.102–0.176	<0.001
Which of the following are not true about follow-up support for individuals who are quitting smoking?	301 (68.3)	414 (93.9)	0.209–0.303	<0.001
**Behavioral counseling Modules 3 and 4**				
The main goals of behavioral counselling are to…	349 (79.0)	419 (94.8)	0.117–0.199	<0.001
There is no evidence that counselling helps patients to quit smoking.	365 (82.6)	429 (97.1)	0.107–0.183	<0.001
Which of the following are true?	385 (87.3)	430 (97.5)	0.070–0.134	<0.001
How long does a craving typically last?	231 (52.3)	426 (96.4)	0.392–0.490	<0.001
The 4Ds strategies are the recommended approach for dealing with cravings to smoke and include.	319 (73.0)	427 (97.7)	-1.397–0.985	0.734
Recent quitters are still vulnerable to relapse, especially in the first three to six months after quitting.	412 (93.2)	428 (96.8)	0.007–0.066	0.016
Motivational interviewing is…	233 (52.8)	394 (89.3)	0.311–0.419	<0.001
The goal of motivational interviewing is not necessarily to get the patients to change but rather to get the patient talking and thinking about changing their smoking behavior.	353 (80.0)	436 (98.6)	0.150–0.226	<0.001
Motivational interviewing techniques clinicians should use, include…	358 (81.0)	431 (97.5)	0.127–0.203	<0.001
Developing discrepancy between a patient’s current behavior and expressed priorities, values and goals is a strategy used as part of motivational interviewing. This can be done by…	180 (40.7)	344 (77.8)	0.117–0.199	<0.001
**Pharmacotherapy Module 5**				
Are nicotine replacement therapies contraindicated for people with cardiovascular disease?	318 (72.6)	426 (97.3)	0.203–0.290	<0.001
Is it safe to continue to smoke while using nicotine replacement therapy?	201 (45.7)	427 (97.0)	0.464–0.563	<0.001
Nicotine replacement therapy should only be used for a maximum of 12 weeks.	244 (55.5)	399 (90.7)	0.299–0.406	<0.001
You may use any form of nicotine replacement therapy alone but they should not be used in combination.	248 (56.5)	431 (98.2)	0.369–0.465	<0.001
A patient smokes 1 pack of cigarettes per day and smokes within 5 minutes of waking in the morning. What dose of NRT should he be started on?	206 (46.8)	342 (77.7)	0.255–0.363	<0.001
A patient is using NRT but reports severe cravings and is concerned he/she may return to smoking. What do you recommend?	244 (55.5)	402 (91.4)	0.307–0.411	<0.001
A woman using Bupropion reports a variety of side effects since beginning the medication. What do you recommend to address this issue?	242 (55.0)	396 (90.0)	0.298–0.402	<0.001
Bupropion is contraindicated among… (Click all that apply)	353 (80.2)	427 (97.0)	0.128–0.208	<0.001
What is the most common side effects of Varenicline?	300 (68.5)	425 (96.2)	0.232–0.325	<0.001
Which of the following strategies can be used for patients who report experiencing nausea while using Varenicline?	229 (52.0)	378 (85.9)	0.280–0.397	<0.001
Patients using Varenicline should be advised to quit smoking how many weeks after using the treatment?	22 (50.9)	339 (77.0)	0.206–0.317	<0.001
For patients using Varenicline who experience vivid dreams it can be recommended that they…	333 (75.9)	428 (97.5)	0.175–0.258	<0.001
It is safe to use Varenicline for 6-month period or longer?	247 (56.3)	371 (84.5)	0.225–0.340	<0.001
Which are the most effective medications in terms of increasing success rates	355 (80.7)	433 (98.4)	0.139–0.216	<0.001

a Multiple choice questions.

* 95% confidence interval of difference between pre- and post-assessments.

** McNemar-Bowker test.

Table 3 presents the changes in healthcare professionals’ self-reported attitudes, normative beliefs, self-efficacy (perceived behavioral control), intentions of delivering tobacco treatment interventions as well as the importance of smoking cessation interventions, before and after the completion of the eLearning curriculum. Favorable changes were documented for attitudes in five out of six areas assessed. Normative beliefs of the participants also changed after the course with statistically significant results in four out of the five statements. A statistically significant increase in healthcare professionals’ self-efficacy to help their patients quit smoking was documented between the pre- and post-intervention assessment (z-score = -13.083; p<0.001).

**Table 3:** Self-reported attitudes, normative beliefs, perceived behavioral control and intention changes of the healthcare professionals that participated in the ENSP eLearning for treating tobacco dependence

*Variables*	*Pre Median (IQR)*	Pre IQR*** 25–75	*Post Median (IQR)*	Post IQR*** 25–75	z-score*	*p*
**Attitudes^a^**						
Counseling by a clinician helps motivate smokers to quit.	5.0 (1.0)	4.0 – 5.0	5.0 (0.0)	5.0 – 5.0	-7.370	<0.001
For many tobacco users smoking is an addiction.	5.0 (1.0)	4.0 – 5.0	5.0 (1.0)	4.0 – 5.0	-2.820	0.005
First line pharmacotherapies for smoking cessation work well in helping patients quit.	3.0 (1.0)	3.0 – 4.0	5.0 (1.0)	4.0 – 5.0	-13.243	<0.001
First line pharmacotherapies for smoking cessation have side effects that outweigh their benefits.	2.0 (2.0)	1.0 – 3.0	1.0 (2.0)	1.0 – 3.0	-4.265**	<0.001
Tobacco use is killing too many people.	5.0 (1.0)	4.0 – 5.0	5.0 (0.0)	5.0 – 5.0	-1.390	0.164
It is my usual practice to assist my patients to quit smoking.	4.0 (2.0)	4.0 – 5.0	4.0 (1.0)	4.0 – 5.0	-5.346	<0.001
**Normative beliefs^a^**						
Smoking cessation is an important part of my role as a healthcare professional.	5.0 (1.0)	4.0 – 5.0	5.0 (0.0)	5.0 – 5.0	-5.316	<0.001
Smoking is a personal decision which does not concern the healthcare professional.	1.0 (2.0)	1.0 – 3.0	1.0 (2.0)	1.0 – 3.0	-0.360	0.719
Healthcare professionals should advise patients to quit smoking even if it is not the reason for the visit.	5.0 (1.0)	4.0 – 5.0	5.0 (0.0)	5.0 – 5.0	-2.562	0.010
Healthcare professionals should make appointments specifically to help patients quit.	5.0 (2.0)	3.0 – 5.0	5.0 (1.0)	4.0 – 5.0	-5.096	<0.001
A patient’s will power alone is what will determine their success with quitting.	4.0 (1.0)	2.0 – 4.0	3.0 (2.0)	2.0 – 4.0	-2.968**	0.003
**Perceived behavioral control (self-efficacy)^a^**						
I have the required skills to help my patients quit smoking.	3.0 (2.0)	2.0 – 4.0	4.0 (1.0)	4.0 – 5.0	-13.083	<0.001
I do not feel I have effective methods to assist my patients with quitting.	3.0 (2.0)	2.0 – 4.0	2.0 (2.0)	1.0 – 3.0	-7.949**	<0.001
My patients follow my advice about behavior change.	3.0 (1.0)	3.0 – 4.0	4.0 (2.0)	3.0 – 5.0	-11.256	<0.001
My patients who smoke want to quit smoking.	3.0 (1.0)	3.0 – 4.0	4.0 (2.0)	3.0 – 5.0	-6.931	<0.001
I know where to refer patients for help with smoking cessation.	4.0 (3.0)	4.0 – 5.0	5.0 (1.0)	4.0 – 5.0	-10.388	<0.001
**Intentions^a^**						
Address tobacco use with all my patients as priority.	4.0 (2.0)	3.0 – 5.0	5.0 (1.0)	4.0 – 5.0	-4.414	<0.001
Document tobacco use status in the patient’s medical record.	5.0 (2.0)	3.0 – 5.0	5.0 (1.0)	4.0 – 5.0	-6.703	<0.001
Offer my support to all my patients making a quit attempt.	5.0 (1.0)	4.0 – 5.0	5.0 (0.0)	5.0 – 5.0	-7.022	<0.001
Provide brief smoking cessation counseling (3–5 minutes).	5.0 (1.0)	4.0 – 5.0	5.0 (0.0)	5.0 – 5.0	-5.586	<0.001
Give my patients written materials about quitting smoking.	5.0 (2.0)	3.0 – 5.0	5.0 (1.0)	4.0 – 5.0	-4.719	<0.001
Discuss available quit-smoking medications with my patients who smoke.	4.0 (2.0)	3.0 – 5.0	5.0 (1.0)	4.0 – 5.0	-9.726	<0.001
Prescribe a quit smoking medication for patients ready to quit.	4.0 (3.0)	2.0 – 5.0	5.0 (1.0)	4.0 – 5.0	-8.345	<0.001
Schedule dedicated appointment to develop quit plans with my patients.	4.0 (2.0)	3.0 – 5.0	5.0 (1.0)	4.0 – 5.0	-7.327	<0.001
Be persistent in addressing tobacco use with my patients even if I am not effective the first time.	5.0 (1.0)	4.0 – 5.0	5.0 (0.0)	5.0 – 5.0	-5.911	<0.001
**Importance of helping patients quit smoking^b^**	10.0 (1.0)	9.0 – 10.0	10.0 (0.0)	10.0 - 10.0	-4.731	<0.001

a On a scale from 1 to 5 (strongly disagree to strongly agree).

b On a scale from 1 to 10.

* Based on negative ranks.

** Based on positive ranks.

*** IQR: interquartile range.

Post period: missing data n=2.

An increase was also documented in the intention to address tobacco use as a priority (z-score = -4.414; p<0.001), document tobacco use (z-score = -6.703; p<0.001), offer support (z-score = -7.022; p<0.001), provide brief counseling (z-score = -5.586; p<0.001), give written material (z-score = -4.719; p<0.001), discuss available medication (z-score = -9.726; p<0.001), prescribe medication for smoking cessation (z-score = -8.345; p<0.001), schedule dedicated appointment to develop a quit plan (z-score = -7.327; p<0.001) and be persistent on addressing tobacco use with the patients even if they are not effective the first time (z-score = -5.911; p<0.001).

## DISCUSSION

Our study evaluated the effectiveness of an accredited eLearning curriculum in increasing key constructs known to be related to tobacco treatment delivery. Results indicated that the eLearning program improved participants’ knowledge, attitudes, beliefs, self-efficacy (perceived behavioral control) and intentions associated with the delivery of evidence-based tobacco treatment interventions. This is important as recent European population-based studies have found that many patients report that they did not receive any advice or support to quit during a healthcare visit^1,12,13^.

Results from a recent systematic review highlighted that digital education was at least as effective as traditional or usual learning, resulting in similar or higher improvement in knowledge and satisfaction, and sometimes in the attitude of the healthcare professionals, following digital education compared with conventional learning^4^. This is an essential aspect as eLearning courses and in general digital education can increase educational opportunities^14^ by providing training and new skills to healthcare professionals who want to integrate tobacco-dependence treatment into their daily clinical practice^15,16^ and at the same time overcome the barrier of distance in healthcare professionals’ education^14^.

Previous results, from our team, also showed that evidence-based training increases healthcare workforce self-efficacy, competence, knowledge and attitudes related to tobacco treatment^7^ and improves the rates at which evidence-based tobacco treatment is delivered to patients^8,9^. Additionally, pre-post studies evaluating the impact of the tobacco treatment guidelines and training for patients with multiple morbidities (CVD, diabetes and COPD) also showed significant increases in physicians’ knowledge and self-efficacy immediately following and at 6 months after exposure to training sessions^10^. Significant increases were also recorded in knowledge, self-efficacy and rates at which doctors delivered evidence-based tobacco treatment following exposure to a live educational intervention^11^. All the above support the findings of our study. However, a follow-up, preferably at 6 months, after the completion of the eLearning course, would be useful to evaluate the sustainability of our results and also the level of the implementation in the healthcare providers’ daily clinical practice.

Previous studies have identified a lack of knowledge and skills among healthcare professionals as a key barrier to the delivery of smoking cessation advice^17^. In addition, due to lack of knowledge, low motivation, perceived low self-efficacy and concerns about inadequate time and workload, many healthcare professionals do not provide effective cessation services^17,18^. However, implementation of tobacco dependence treatment for healthcare professionals has been shown to increase their cessation efforts significantly with their patients and quit attempts^19,20^, as well as improve trainees’ attitudes and perceived behavioral control in providing tobacco dependence services^21^. The importance of continuing medical education training programs to address this gap in knowledge and skills has also been acknowledged and can significantly impact healthcare professional competence, future clinical practice and patient outcomes if embedded into the healthcare system^22-26^. Based on all the aforementioned, time and distance saving and cost-effective training programs that provide new knowledge and skills are essential to ensure that healthcare professionals are confident to deliver smoking cessation interventions to all their patients who smoke.

### Strengths and limitations

Previous research that has evaluated digital education for smoking cessation until now has mainly focused on patient interventions^27,28^. In contrast, our research has focused on training healthcare professionals. We present a novel approach by using a pre-post design to monitor the change at the participant level for a broad representation of the healthcare professionals from many European countries.

However, as the eLearning course was accredited, there were some restrictions on the procedure of the completion of the course, as participants had to successfully complete each module’s test to receive accreditation. In addition, the profile of the participants and the voluntary nature of our course may mean that the participants were more motivated than the general population of healthcare professionals. On the other hand, for the same reasons, the course healthcare professionals may have over-performed in their responses for changing attitude norms, perceived behavioral control and intentions in delivering tobacco treatment interventions with their patients who smoke. For these reasons, the generalizability of our findings requires further investigation.

## CONCLUSIONS

The ENSP eLearning intervention, built on evidence-based tobacco treatment eLearning courses, was associated with significant increases in healthcare professional’s knowledge, attitudes, normative beliefs, perceived behavioral control and intention to integrate tobacco treatment delivery into daily clinical practice. Future research should examine supplementary methods for supporting the broader dissemination of well-designed digital interventions and eLearning programs, and develop targeted strategies for all healthcare professionals.
